# NMD inhibition fails to identify tumour suppressor genes in microsatellite stable gastric cancer cell lines

**DOI:** 10.1186/1755-8794-2-39

**Published:** 2009-06-29

**Authors:** Tineke E Buffart, Marianne Tijssen, Jamila El-Bchiri, Alex Duval, Mark A van de Wiel, Bauke Ylstra, Gerrit A Meijer, Beatriz Carvalho

**Affiliations:** 1 Dept Pathology, VU University Medical Center, Amsterdam, The Netherlands; 2 INSERM U434, Hôpital Saint-Louis, Paris, France; 3 Dept Mathematics, VU University Medical Center, Amsterdam, The Netherlands; 4 IPATIMUP, Department of Pathology, Medical Faculty, Hospital de S. João, Porto, Portugal

## Abstract

**Background:**

Gastric cancers frequently show chromosomal alterations which can cause activation of oncogenes, and/or inactivation of tumour suppressor genes. In gastric cancer several chromosomal regions are described to be frequently lost, but for most of the regions, no tumour suppressor genes have been identified yet. The present study aimed to identify tumour suppressor genes inactivated by nonsense mutation and deletion in gastric cancer by means of GINI (gene identification by nonsense mediated decay inhibition) and whole genome copy number analysis.

**Methods:**

Two non-commercial gastric cancer cell lines, GP202 and IPA220, were transfected with siRNA directed against *UPF1*, to specifically inhibit the nonsense mediated decay (NMD) pathway, and with siRNA directed against non-specific siRNA duplexes (CVII) as a control. Microarray expression experiments were performed in triplicate on 4 × 44 K Agilent arrays by hybridizing RNA from *UPF1*-transfected cells against non-specific CVII-transfected cells. In addition, array CGH of the two cell lines was performed on 4 × 44K agilent arrays to obtain the DNA copy number profiles. Mutation analysis of GINI candidates was performed by sequencing.

**Results:**

*UPF1 *expression was reduced for >70% and >80% in the GP202 and IPA220 gastric cancer cell lines, respectively. Integration of array CGH and microarray expression data provided a list of 134 and 50 candidate genes inactivated by nonsense mutation and deletion for GP202 and IPA220, respectively. We selected 12 candidate genes for mutation analysis. Of these, sequence analysis was performed on 11 genes. One gene, *PLA2G4A*, showed a silent mutation, and in two genes, *CTSA *and *PTPRJ*, missense mutations were detected. No nonsense mutations were detected in any of the 11 genes tested.

**Conclusion:**

Although *UPF1 *was substantially repressed, thus resulting in the inhibition of the NMD system, we did not find genes inactivated by nonsense mutations. Our results show that the GINI strategy leads to a high number of false positives.

## Background

As many other solid tumours, gastric cancer develops through an accumulation of genetic and epigenetic alterations. Although the knowledge of genetic and epigenetic events occurring in gastric cancer is increasing, it is still far from being complete.

Two major types of genetic instability are described in gastric cancer, chromosomal instability and microsatellite instability[1]. Chromosomal instable tumours show gross chromosomal abnormalities leading to loss and or gain of large genomic areas, while microsatellite instable tumours show an increased mutation rate at the nucleotide level and in general do not show gross chromosomal abnormalities. The majority of gastric cancers have a chromosomal instable phenotype and many studies have been published describing frequent occurrence of chromosomal aberrations in gastric cancers [2-11]. Chromosomal alterations can cause activation of oncogenes, by increasing the copy number, and/or inactivation of tumour suppressor genes, by loss of alleles. In case of tumour suppressor genes, usually both alleles must be inactivated in order to abrogate the function of a gene, which can be achieved by any combination of loss, mutation, or promoter hypermethylation. In gastric cancer several chromosomal regions have been described to be frequently lost[6,11,12], but in most of these regions, no tumour suppressor genes have been identified yet.

In eukaryote cells, mRNAs molecules that contain premature termination codons (PTCs) due to nonsense mutations are detected and rapidly degraded by the nonsense-mediated decay (NMD) mechanism. NMD is mediated through the assembly of protein complex coded by genes such as the ones belonging to the UPF family e.g. *RENT-1/UPF1*, *RENT-2/UPF2*, *UPF-3A*, and *UPF-3B*[13]. RENT-1/UPF1 has been shown to play a crucial role in the function of the NMD system. Taking advantage of the existence of this regulatory system in the cells, Noensi and Dietz described a strategy, called GINI (Gene Identification by Nonsense-mediated decay Inhibition), to identify tumour suppressor genes harbouring premature stop-codons[14]. Microarrays are used to identify potential nonsense transcripts that are increased in abundance after inhibition of the NMD system, by comparing the sample to itself after inhibition of NMD. The NMD pathway can be pharmacologically blocked by treating the cells with a translation inhibitor, such as emetine, resulting in stabilization of mutated transcripts containing a premature stop-codon. However, this drug also induces a stress response resulting in increased mRNA levels of many transcripts. To more specifically inhibit the NMD pathway, a different strategy has been described in which a siRNA directed against *UPF1 *is used[15,16].

The combination of NMD microarray data on putative nonsense mutations with array CGH data on deleted genomic areas enables the detection of biallelic inactivation events of tumour suppressor genes, as shown previously in prostate cancer[17]. Therefore, the present study aims to identify tumour suppressor genes inactivated by nonsense mutation and deletion in gastric cancer by means of GINI and whole genome DNA copy number analysis.

## Methods

### Cell lines and cell culture

Two non-commercial gastric cancer cell lines, GP202 and GP220, established and characterized in IPATIMUP, Porto[18] were used for siRNA transfection. These particular cell lines were derived from two different patients and were not immortalized by viral infection. The cell lines were maintained in RPMI supplemented with 10% fetal calf serum, 100 U/mL penicillin, 100 μg/mL streptomycin and 2 mmol/L L-glutamine (Life Technologies, Breda, NL).

### DNA isolation and Array CGH

Genomic DNA was isolated using TRIzol reagent (Invitrogen, Breda, NL) according to the manufacturer's protocol with some modifications . DNA isolated from blood obtained from eighteen healthy males was pooled and used as normal reference. DNA concentrations were measured on a Nanodrop ND-1000 spectrophotometer (Isogen, IJsselstein, NL). 500 ng of DNA was labelled using the Enzo Genomic DNA Labelling kit as described previously (Enzo Life Sciences, Farmingdale, USA)[19]. Hybridizations were performed on slides containing four arrays, with each array containing 45220 in-situ synthesized 60-mer oligonucleotides, representing 42494 unique chromosomal locations (Agilent Technologies, Palo Alto, USA)[19].

Images of the arrays were acquired using a microarray scanner G2505B (Agilent technologies) and image analysis was performed using feature extraction software version 9.5 (Agilent Technologies,). The Agilent CGH-v4_95 protocol was applied using default settings. Oligonucleotides were mapped according to the human genome build NCBI 35 (May 2004). For both Cy3 and Cy5 channels, local background was subtracted from the median intensities. The log_2 _tumour to normal ratio was calculated for each spot and normalized against the median of the ratios of all autosomes.

### UPF1 siRNA transfection

Transfection of both cell lines was performed in 35 mm dishes with 100 nM of siRNA duplexes directed against *UPF1 *(Dharmacon, Chicago, IL) or non-specific siRNA duplexes (CVII) (Dharmacon) using the lipofectamine 2000 reagent (Invitrogen) according to the manufacturer's instructions. Cells were collected for total RNA extraction 72 h after transfection. Each transfection experiment was performed in duplicate on three different days.

### RNA isolation procedures and quantitative real-time PCR

Total RNA was extracted from each cell line using the RNeasy kit (Qiagen, Westburg, Leusden, NL) including a DNase digestion step, according to the manufacturer's instructions. Concentrations were measured on a Nanodrop ND-1000 spectrophotometer (Isogen). Synthesis of cDNA was performed with random primers using the high capacity cDNA Archive Kit kit (Applied Biosystems). SDS 2.1 Applied Biosystems analysis software was used to determine the Ct number at which increase in signal is associated with exponential amplification of the PCR products, needed to quantify the expression values. Quantification of the 18S ubiquitous RNA was used as the endogenous reference. The delta Ct was determined in each case by subtracting the average Ct value of the target gene from the average Ct value of the 18S gene. The percentage of inhibition of the *UPF1 *gene was calculated by subtracting the mean delta ct of the *UPF1 *siRNA transfected cells by the mean delta ct of the CVII siRNA control transfected cells, as previously described[15].

### Microarray expression analysis

Microarray expression experiments were performed on 4 × 44 K Agilent expression arrays (Agilent technologies) by hybridizing *UPF1 *siRNA transfected cells against non-specific CVII siRNA control transfected cells, according to the manufacturer's instructions. RNA quality was evaluated by generating an electropherogram on the Agilent Bioanalyzer 2100 using a RNA 6000 Nano-LabChip (Agilent Technologies). RNA integrity numbers (RIN) of >9.0 were considered as good quality RNA. Experiments were performed in dye-swap and in triplicate, resulting in six arrays per cell line. Images of the arrays were acquired using a microarray scanner G2505B (Agilent technologies) and image analysis was performed using feature extraction software version 9.5 (Agilent Technologies). The Agilent GE2-v5_95 protocol was applied using default settings.

All data pre-processing and analysis was performed using the R-Bioconductor package Limma[20]. First, a robust Edwards background correction was applied, followed by within-array and between-array normalization using loess and scale standardization, respectively. Differential expression between *UPF1 *siRNA transfected cells and non-specific CVII siRNA control transfected cells was assessed by use of a linear model, which accounts for a blocking factor, the day effect (triplicate). Moreover, the two-sample t-statistic modified for correlation between the two duplicates was used. Finally, p-values were adjusted for multiple testing using convential Benjamini-Hochberg FDR correction.

Array CGH and microarray expression data can be assessed using the Gene Expression Omnibus (GEO) , under the accession number GSE12928.

### Mutation analysis

From each RNA sample, 1 μg was reverse transcribed to cDNA using oligo(dT)_20 _Primer (Invitrogen) with AMV reverse transcriptase (Promega, Leiden, NL). Mutation screening involved the entire coding region using primers overlapping the exon-exon boundaries. Each reaction was carried out in a total volume of 25 μl containing 1 μl of cDNA, 1,5 μl MgCl_2 _(25 mM), 2.5 μl dNTPs (2 mM), 1.25 Units of Amplitaq Gold polymerase (Applied Biosystems), 2.5 μl GeneAmp^®^10× PCR buffer II and 12.5 pmol for each forward and reverse primer. When DMSO was added to the reaction, 2.5 Units of Amplitaq Gold polymerase was used. Amplification conditions were an initial denaturation step of 5 minutes at 94°C followed by 40 cycles of 30 seconds at 94°C, 30 seconds at 55–57°C (depending on the primer pair), 30 seconds at 72°C, and ending with 7 minutes at 72°C. PCR products were evaluated in a 2% agarose gel. PCR products were purified using Shrimp Alkaline Posphatase and Exonuclease (SAP and EXO enzymes) (USB corporation, Cleveland, USA) to remove the phosphate groups from the excess dNTPs left over from the PCR reaction and to digest single stranded PCR primers into dNTPs by incubating for 30 minutes at 37°C, followed by a 15 minute incubation at 80°C to inactivate the enzymes. Sequence reactions were performed in a total volume of 10 μl containing 3.5 μl purified PCR product, 2 μl sequencing buffer (5×), 0.5 μl BigDye Terminater v3.1 mix (Applied Biosystems) and 10 pmol of each forward and reverse primer. Amplification was performed in 25 cycles of 30 seconds at 96°C, 15 seconds at 45°C and 4 minutes at 60°C. Samples were precipitated by 0.1 volume NaAc (3 M; pH 5.3) and 2.5 volume ethanol. Sequencing of the PCR products was performed in 10 μl deionised formamide (Applied Biosystems) on an ABI 3130 capillary sequencer (Applied Biosystems). Sequence analysis was carried out using the sequence Analysis 5.2 software (Applied Biosystems) and the Vector NTI software (Invitrogen). Details of the primer sequences, annealing temperatures and extra PCR conditions are given in Additional file 1.

Genomic DNA sequence analysis was performed with new designed primers by BaseClear (Leiden, The Netherlands).

## Results

### Array CGH profiles

**Figure 1:**
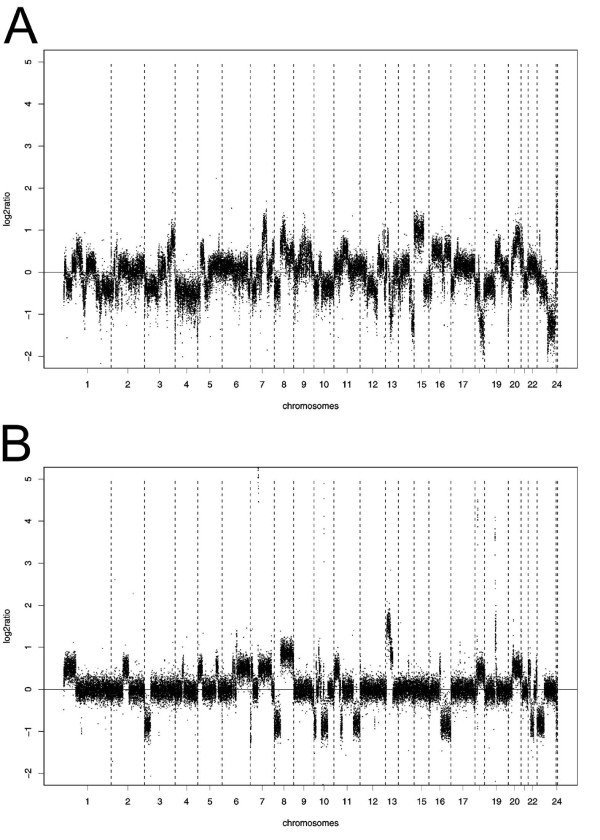
**DNA copy number profile obtained by array CGH analysis of the GP202 gastric cancer cell line**. **(A) and the IPA220 gastric cancer cell line (B).** Normalised log_2 _tumour to normal ratios of every spot are presented sorted by position in chromosomal order (1-Y). Dashed-vertical lines – transition between the chromosomes.

Array CGH profiles of the cell lines GP202 and IPA220 were obtained to detect the deleted areas potentially harbouring tumour suppressor genes. Array CGH profiles of the GP202 and IPA220 gastric cancer cell lines are shown in figure 1A and 1B, respectively. A detailed overview of all gains and losses detected in these two cell lines is given in table 1 and 2.

**Table 1:** Overview of all chromosomal gains and losses detected in the gastric cancer cell line GP202

**Gains**			**Losses**		
**Cytoband**	**Start (bp)**	**Segment size (Mb)**	**Cytoband**	**Start (bp)**	**Segment size (Mb)**
			1p36.33-1p36.32	604268	3.10
			1p36.21-1p35.1	14554092	18.26
1p32.3-1p31.2	51415638	17.20			
1p31.1-1p31.1	75490653	3.21			
1p31.1-1p22.3	84414481	2.76			
			1p22.2-1p13.3	91093183	19.83
			1q24.3-1q44	167792143	77.63
			2p25.3-2p23.3	29193	24.75
			2p23.1-2p22.2	30171297	7.64
2p22.2-2p22.1	38068378	1.57			
			2p22.1-2p21	39804095	2.17
2p21-2p21	42322892	1.85			
			2p21-2p16.2	44568947	8.24
			2p12-2p12	75641228	7.06
			2q14.1-2q14.1	114390667	2.40
			2q24.1-2q24.1	155514824	1.07
			2q24.3-2q24.3	166690618	1.25
			2q32.1-2q32.2	184949202	4.73
			3p26.3-3p11.1	224727	87.69
			3q11.2-3q11.2	95295727	3.77
3q12.1-3q12.2	100024301	1.70			
			3q22.3-3q24	140145815	8.85
3q24-3q25.1	149729642	3.28			
3q25.2-3q26.1	156397505	6.15			
			3q26.1-3q26.1	162557800	6.25
3q26.2-3q26.31	170346051	4.88			
3q26.32-3q29	177640401	20.35			
			4p16.3-4q35.2	62447	191.20
			5p15.33-5p14.1	148243	29.04
5p13.3-5p12	30636882	13.08			
5q11.2-5q12.1	50714334	8.21			
			5q12.3-5q14.2	65345185	17.43
			5q31.3-5q31.3	143269803	1.01
5q33.3-5q34	158674495	4.67			
			6p21.2-6p21.2	38554812	0.15
			6p12.1-6p12.1	54055870	2.14
			6p11.2-6q13	58122432	12.68
			6q15-6q16.1	91282705	5.81
			6q16.3-6q21	101156415	4.21
			6q24.3-6q24.3	147629943	0.12
6q25.2-6q25.3	155542493	0.97			
			6q25.3-6q27	156677778	14.13
			7p21.3-7p14.1	8408484	33.10
7p11.2-7p11.2	54921296	0.57			
			7q11.21-7q11.22	65833193	4.96
			7q11.23-7q11.23	76818583	0.16
7q21.13-7q31.1	90482514	19.64			
7q31.1-7q31.1	110637986	1.28			
			7q31.1-7q31.2	113074003	2.15
			7q31.33-7q31.33	124912671	0.76
7q34-7q35	142863066	0.23			
7q35-7q36.3	147112584	11.48			
			8p23.3-8p23.1	181530	11.15
			8p23.1-8p11.21	12627630	29.40
8p11.21-8q21.3	42137357	49.48			
8q22.1-8q22.3	95005574	9.49			
8q24.11-8q24.3	117851748	28.40			
9p24.3-9p24.2	204367	3.94			
			9p23-9p22.3	9335062	5.76
			9p21.1-9p21.1	28203365	2.53
9q13-9q21.13	68264900	4.61			
9q21.13-9q21.31	74400503	5.06			
9q21.33-9q22.1	85372508	2.87			
9q22.2-9q22.31	89232280	2.91			
9q22.31-9q22.32	93160028	1.30			
9q22.33-9q31.1	97586481	3.58			
9q31.1-9q31.1	104314825	0.46			
9q31.3-9q32	110265680	3.46			
9q33.1-9q33.2	115059550	7.40			
9q33.2-9q33.3	122895073	0.42			
9q33.3-9q34.11	126436029	3.69			
			10p15.3-10p11.22	138206	34.13
			10q21.1-10q26.3	54480789	80.81
11p11.2-11p11.2	43422214	1.84			
11q12.1-11q13.4	57256449	13.13			
			11q14.3-11q14.3	87970040	0.76
			11q14.3-11q14.3	89525497	0.05
			11q21-11q22.1	96058479	3.39
			12q12-12q22	37052371	55.79
12q23.1-12q23.3	96894351	6.93			
12q24.23-12q24.23	117761811	0.61			
12q24.31-12q24.31	121971290	1.03			
			12q24.31-12q24.33	123020939	9.36
13q12.2-13q13.1	27392825	5.14			
			13q13.3-13q21.33	34631933	36.49
			13q31.1-13q31.3	79911534	13.43
			14q11.2-14q11.2	19365051	2.79
			14q11.2-14q12	22545671	6.72
			14q13.3-14q21.1	36741208	0.35
			14q21.1-14q21.2	40137052	3.71
			14q21.2-14q21.3	45739681	3.13
			14q31.1-14q32.33	78503451	27.83
15q11.2-15q24.1	19109124	52.29			
			15q24.1-15q26.3	71444303	28.72
			16p13.3-16p13.3	1339391	3.35
16p13.3-16p12.3	5565717	13.83			
			16p12.3-16p12.3	19426488	0.04
16p12.3-16q21	19493912	38.95			
			16q22.1-16q22.1	66730760	1.00
16q22.1-16q23.1	67757442	9.01			
16q23.1-16q24.3	77573211	11.08			
			17p13.3-17p13.1	48539	7.48
17q11.1-17q11.2	22335103	1.75			
			18p11.32-18q12.1	170229	30.01
			18q12.3-18q23	36319629	39.76
			19p13.3-19q12	232080	33.93
19q12-19q13.31	36336102	12.74			
			19q13.32-19q13.32	52132977	0.56
			20p13-20p12.1	18580	14.51
20p12.1-20p12.1	14772372	1.30			
20p11.21-20q13.33	24262140	38.10			
21q21.1-21q21.3	17434961	11.95			
			21q21.3-21q22.3	29452714	16.38

**Table 2:** Overview of all chromosomal gains and losses detected in the gastric cancer cell line IPA220.

**Gains**			**Losses**		
**Cytoband**	**Start (bp)**	**Segment size (Mb)**	**Cytoband**	**Start (bp)**	**Segment size (Mb)**
1p33-1p36.33	1532086	45.95			
1p33-1p33	48905659	0.05			
			1p22.3-1p22.3	85435027	0.06
2q22.1-2q11.1	95057834	45.61			
2q33.1-2q33.1	200167066	0.43			
			3p26.3-3p22.1	224727	40.68
			4p16.3-4p16.3	62447	1.83
4q13.3-4q13.1	60036497	14.87			
5p12-5p15.33	148243	43.37			
5q33.2-5q31.3	141986337	12.82			
			6p25.3-6p25.3	204528	0.03
6p12.3-6p21.1	43716420	5.79			
6q12-6p12.1	56790132	12.18			
6q27-6q14.1	80423355	90.39			
			7p22.3-7p22.1	149268	4.55
7p21.1-7p22.1	4874872	14.88			
7q34-7p12.1	53246250	86.37			
			8p23.3-8p11.21	181530	42.99
8q24.3-8q11.1	47062121	99.19			
			9q34.2-9q34.3	133008166	5.28
			10p15.3-10p13	138206	16.20
10p12.33-10p13	16543648	1.39			
10q11.22-10p11.22	33177446	13.39			
10q11.23-10q11.23	51219561	0.04			
			10q21.1-10q22.1	53701099	18.43
10q22.1-10q22.1	74316662	0.24			
			10q22.1-10q22.2	74569446	0.95
10q22.2-10q22.2	75567726	0.57			
			10q22.2-10q22.2	76194936	0.41
			10q22.2-10q24.2	77212707	23.02
			10q26.3-10q26.3	133641790	1.65
11p14.1-11p15.5	2389958	27.82			
			11p12-11p11.12	39023903	11.61
11q13.1-11q13.1	65527252	0.02			
			11q14.3-11q14.3	88320337	0.38
			11q22.3-11q23.3	103942362	13.29
			11q23.3-11q25	117574530	16.38
12p13.33-12p13.33	1471571	0.32			
13q21.31-13q12.13	24807238	37.57			
14q24.3-14q24.3	74237320	1.26			
			14q32.33-14q32.33	103705675	2.24
			16p13.3-16p13.3	258880	1.09
16q12.2-16q12.1	48880404	2.95			
			16q12.2-16q22.1	53523174	12.50
			16q22.1-16q24.3	66132070	22.52
17q21.31-17q21.31	41566540	0.06			
18q11.2-18p11.22	10662792	7.80			
			18q11.2-18q11.2	20896260	0.38
18q23-18q11.2	21531881	53.70			
18q23-18q23	75803559	0.28			
19p13.3-19p13.3	232080	0.75			
19q13.12-19q12	34074193	6.56			
20p11.21-20p11.22	21329267	4.35			
20q13.33-20q11.21	29352138	33.01			
21q21.1-21q11.2	13926078	6.84			
			21q22.3-21q22.3	45354820	1.07
22q11.22-22q11.1	14433473	7.35			
22q12.1-22q11.23	23692593	2.23			
			22q12.1-22q13.1	26016519	10.56
			22q13.1-22q13.1	37683612	0.03
22q13.33-22q13.31	44867938	3.71			

### *UPF1 *inhibition and expression array analysis

Using the siRNA strategy, *UPF1 *expression was repressed by >70% for the GP202 gastric cancer cell line (73% 74% and 71% for the biological replicates), and >80% for the IPA220 gastric cancer cell line (82%, 86% and 84% for the biological replicates).

Micoarray expression array analysis yielded 540 spotted oligonucleotides significantly upregulated (adjusted p-values < 0.05) with a log_2 _ratio > 0.7 in the GP202 cells transfected with *UPF1 *siRNA compared to non-specific CVII siRNA control transfected cells. Of these, 164 oligonucleotides, representing 134 different genes, were located in deleted areas. The IPA220 *UPF1 *siRNA transfected cells showed 265 spotted oligonucleotides significantly upregulated (adjusted p-values < 0.05) with a log_2 _ratio > 0.7 compared to non-specific CVII siRNA control transfected cells. Of these, 50 different genes were located in deleted areas. Of these genes, we selected genes with only one known transcript according to ensemble  and genes of which no alternative splice patterns were known. This yielded a list of 10 candidate genes to be inactivated by nonsense mutation and deletion. The genes *PLA2G4A, BMP5, MMP6, KNNMB4 *and *DYM *were candidates for the GP202 gastric cancer cell line and the genes *TXNL4B, FOXK1, PTPRJ*, and *SNN *were candidates for the IPA220 gastric cancer cell line. The gene *SLITRK6 *was selected as a candidate gene for both gastric cancer cell lines, however only in the GP202 gastric cancer cell line this gene was located in a deleted area. In addition we selected two genes potentially inactivated by nonsense mutation which were located outside deleted areas, but showed high log_2 _ratios and no known splice variants (*CSTA *for the GP202 and *INHBB *for the IPA220 gastric cancer cell lines). Candidate genes, including their chromosomal location are presented in Table 3.

**Table 3:** List of 12 candidate genes putatively inactivated by nonsense mutation, including their chromosomal location, the cell line where it was identified and a short description of the gene.

**Gene**	**Location**	**Cell line**	**Description**
PLA2G4A	1q31.1	GP202	phospholipase A2, group IVA
INHBB	2q14,2	IPA220	Inhibin, beta B
CSTA	3q21.1	GP202	Cystatin A
BMP5	6p12.1	GP202	Bone morphogenetic protein 5
MMP6	7p15.3	GP202	membrane protein, palmitoylated 6 (MAGUK p55 subfamily member 6)
FOXK1	7p22.1	IPA220	Forkhead box K1
PTPRJ	11p11.2	IPA220	Protein tyrosine phosphatase, receptor type J
KCNMB4	12q15	GP202	Potassium channel, calcium-activated, large conductance, subfamily M, beta member 4
SLITRK6	13q31.1	GP202, IPA220	SLIT and NTRK-like family, member 6
SNN	16p13	IPA220	stannin
TXNL4B	16q22.2	IPA220	Thioredoxin-like 4B
DYM	18q21.1	GP202	dymecelin

### Mutation analysis

Of the 12 candidate genes, we successfully completed sequence analysis of 11 genes. After complete sequencing, *KCNMB4*, *BMP5*, *DYM*, *TXNL4B*, and *SNN *did not show any mutation. *SLITRK6 *did not show a mutation in the UPF1 siRNA-transfected IPA220 and GP202 gastric cancer cells. However, in the *UPF1 *siRNA-transfected GP202 gastric cancer cells, the first 196 bp of the coding sequence was missing due to PCR failures. For the gene *INHBB *the first 413 bp of the coding sequence was missing due to PCR failures but no mutation was detected in the remaining coding sequence. Due to the PCR failures, sequence analysis of these two genes was also performed on genomic DNA with new designed primers (BaseClear, Leiden, The Netherlands). Although sequencing of *SLITRK6 *was successfully performed, no mutations were detected in this gene. Sequencing the genomic DNA of the gene *INHBB *was unsuccessful, consistent with the cDNA sequence of the gene.

**Figure 2:**
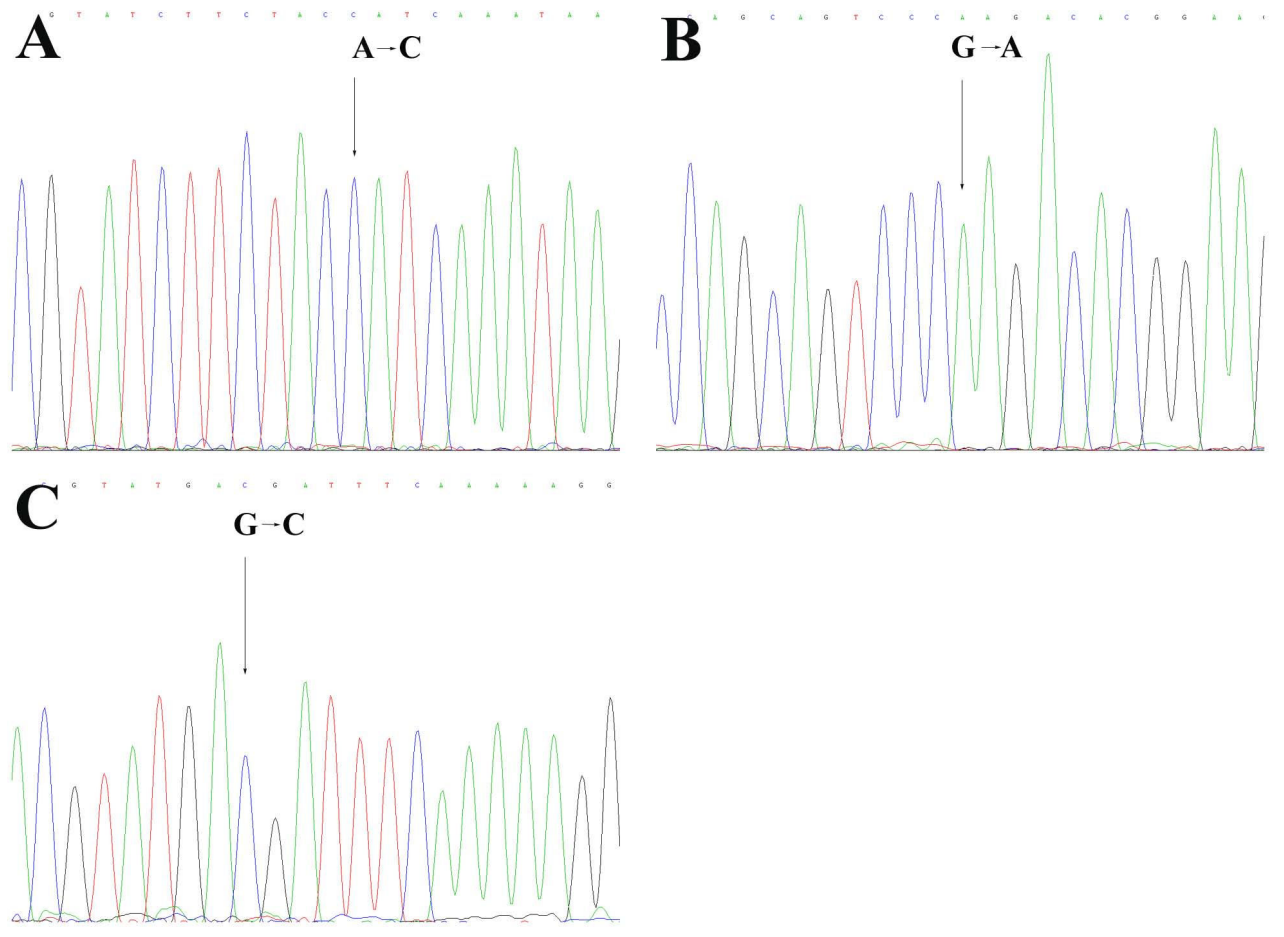
**Mutations analysis of *PTPRJ *in the IPA220 siRNA transfected cells yielded three polymorphisms, A1183C on exon 5 (A), G1333A on exon 6 (B) and G2972C on exon 13 (C)**.

In *CSTA *a heterozygous mutation was detected in both GP202 and IPA220 gastric cancer cells transfected with *UPF1 *siRNA, at position 298 (mRNA seq. NM005213.3) resulting in a G to C substitution, which in turn resulted in an amino acid change from GTA (Valine) to a ATA (Isoleucine) at position 57 of the CSTA protein. In *PLA2G4A*, a mutation was detected in the *UPF1 *siRNA-transfected GP202 gastric cancer cells, at position 1012 (mRNA seq NM_024420.1) resulting in a C to T substitution at position 303 of the protein, but this mutation did not result in a different amino acid (GAC to GAT (Aspartate)). In *PTPRJ*, three mutations were detected in the *UPF1 *siRNA-transfected IPA220 gastric cancer cells. The first mutation resulted in a C to A substitution at position 1183 (mRNA seq NM_002843.3), which resulted in a CAA (glutamine) to CCA (proline) amino acid change at position 276 of the PTPRJ protein. The second mutation resulted in a G to A substitution at position 1333 (mRNA seq NM-002843.3), resulting in an amino acid change from CGA (Arginine) to CAA (Glutamine) at position 326 of the protein. The last mutation resulted in a G to C substitution at position 2972 (mRNA seq (NM_002843.3), resulting in CAG (Glutamine) to GAC (Aspartate) amino acid change at position 836 of the protein (Figure 2). Only the last mutation was detected in the GP202 gastric cancer cells transfected with *UPF1 *siRNA, but this was a heterozygous mutation.

**Figure 3:**
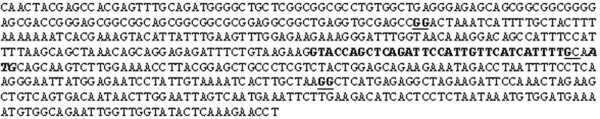
**First part of the mRNA sequence of the MMP6 gene**. The sequence in bold represents the missing sequence in the smaller PCR product, located upstream of the start codon. Bold and underlined nucleotides represent the exon-exon boundaries The start codon ATG is indicated in bold and italic.

Using the first primer set of *MPP6*, two bands were detected on the gel. After sequencing both bands we observed a 33 bp deletion before the coding start site in the shorter band which is suggestive for a splice variant since the exon-exon boundary is involved (Figure 3). No mutations were detected. An overview of the mutations detected in this study is presented in table 4.

**Table 4:** Overview of mutations detected in the candidate genes

**Gene**	**Nucleotide**	**Amino acid**	**Exon**
*CSTA*	G298C	Val57Ile	2
*PLA2G4A*	C1012T	Asp303Asp	15
*PTPRJ*	A1183C	Gln276Pro	5
	G1333A	Arg326Gln	6
	G2972C	Glu836Asp	13

## Discussion

Gastric cancer is a major cause of cancer death, but knowledge about the biology underlying gastric cancer development is still limited. Several chromosomal regions have been described to be frequently deleted in gastric cancer, but in most of the regions, no tumour suppressor genes have been described yet. Using the GINI strategy in combination with array CGH, we aimed to identify tumour suppressor genes inactivated by nonsense mutation and deletion in gastric cancer cell lines.

The first GINI strategies used a translation inhibitor to block the NMD pathway. By using translation inhibitors, the half-lives of many mRNAs are increased making it difficult to identify genes of which mRNA is increased due to the existence of a PTC[21]. In an attempt to improve detection of changes in decay rates, actinomycin D treatment, which stops initiation of mRNA synthesis, has been combined with translation inhibitors[17]. This strategy proved not to be as efficient as initially thought, probably due to side-effects of the drugs which have been suggested to include stabilization of the transcriptome, resulting in protection of the transcripts from degradation. In addition, using drug treatment, an overall stress response is induced resulting in upregulation of many transcripts[21,22]. We have previously used a combination of emitine and actinomycin D treatment to inhibit the NMD machinery in three colorectal cancer cell lines, two microsatellite stable (HT29 and colo205) and one microsatellite instable (RKO). Sequence analysis of the candidate genes did not lead to the identification of any truncation mutation (data not shown).

In the present study, we used siRNAs directly targeting *UPF1 *which plays a central role in the NMD machinery. This approach was thought to result in less false positive genes compared to chemical translation inhibitors[15,22]. However, as the present study indicated, in our hands this method also resulted in multiple false positive candidate genes.

Nonetheless, the GINI approach, using drug translation inhibitors in combination with transcription blockers, has been successfully applied in prostate and colon cancer cell lines[17,23]. Also a different GINI approach (GINI2), using caffeine for NMD inhibition, has been successfully applied in the identification of bi-allelic inactivating mutations[24]. Blocking the NMD machinery using the siRNA strategy was also successful in detecting genes carrying PTCs in colorectal cancer cell lines[15]. However, in all these studies only cancer cell lines with microsatellite instability were analyzed, increasing the chance of success as microsatellite instable cell lines present, due to their phenotype, a high frequency of frameshift mutations leading to PTCs. To our knowledge, this is the first study describing the GINI technology in microsatellite stable gastric cancer cell lines blocking the NMD mechanism by siRNAs targeting *UPF1*. After sequencing the mRNA transcripts of the putative candidate genes, we did not detect nonsense mutations. The fact that the cell lines used in this study are not microsatellite instable may justify the lack of success on finding genes harbouring premature PTCs. However, the technique should also be applicable on microsatellite stable cancer cell lines as shown by Pinyol et al[25]. In their study, five mantle cell lymphoma cell lines were examined which may result in a more accurate and stringent selection of candidate genes compared to our data analysis in which we only used two cell lines.

Although no nonsense mutations were found in this study, we did detect silent or missense mutations in three genes. A silent mutation was detected in the gene *PLA2G4A *at position 303 of the protein. This polymorphism has not been previously described. A missense mutation was detected in the *CTSA *gene at position 57 of the protein changing a Valine into an Isoleucine. In the gene *PTPRJ*, three missense mutations were detected in IPA220 gastric cancer cell line. The mutations Gln276Pro and Arg326Gln in exons 5 and 6, respectively, have been described before as being polymorphisms[26]. To our knowledge, the third mutation, Glu836Asp, in exon 13, has not been previously described, thus the biological consequence, if any, is not clear. Loss of heterozygosity (LOH) of *PTPRJ *has been detected in breast, lung and colon cancers and expression has been shown to induce differentiation and to inhibit growth of breast cancer cells, indicating a function as tumour suppressor gene [26-28]. Since we detected missense mutations of this gene in the gastric cancer cell lines analyzed, it gives the indication that *PTPRJ *might play a role as tumour suppressor gene in gastric cancer, however, apparently not by inactivation by nonsense mutation.

One explanation why our strategy of GINI combined with DNA copy number profiling failed to detect new tumour suppressor genes with nonsense mutations could be that these nonsense mutations are less common than expected. Indeed, recent massive sequencing studies have identified that many cancer related genes often are mutated in only low frequencies, while alternative mechanisms of inactivation like promoter hypermethylation are much more common[29,30].

An important point is why siRNA inhibition of *UPF1 *generates so many false positive hits. Besides a crucial role of *UPF1 *in RNA degradation pathways, the gene also plays a role in DNA replication during the S phase of the cell cycle, and has been shown to be involved in DNA metabolism. UPF1 depletion causes cells to arrest in the S phase and those cells are able to initiate but not complete DNA replication. UPF1 depleted cells have been shown to harbour increased chromatid and chromosome breaks leading to chromosomal aberrations[31,32]. In addition, UPF1 has been shown to promote the rate and efficiency of translation of normal mRNAs in mammalian cells[33]. Translation termination in *UPF1 *mutants was shown to be dependent on the sequence context. Efficient translation termination was observed when UCC (Serine) was located upstream and GCA (Alanine) was located downstream of the PTC. Location of CAA (Glutamine) on either side of the PTC resulted in up to 100 fold reduction in efficiency of translation termination[34]. We could speculate that by inhibiting *UPF1 *by siRNAs also mRNAs without PTCs are not efficiently translated into proteins, and consequently negative feedback loops are not activated, causing the cell to produce more mRNAs due to lack of functional proteins essential for the cell. This in turn can cause accumulation of mRNA resulting in false positive genes. Another hypothesis possibly contributing to the false positive genes found in our analysis can involve the coding sequence surrounding the PTCs which can determine the efficiency of the NMD machinery. Also, NMD downregulates wild-type transcripts and regulates the expression of many physiological transcripts[16]. Physiological substrates for NMD include transcripts with alternative splice variants. For this reason we excluded the genes with multiple known splice variants as candidate genes carrying a PTC, thereby limiting the rate of false positive candidate genes. Nonetheless, among the genes with one known transcript, the GINI technology still yielded many false positive candidate genes.

Finally, although 70–80% depletion of *UPF1 *is thought to be sufficient for inhibiting the NMD machinery both in microsatellite instable cell lines[15] as well as in microsatellite stable gastric cancer cell lines[35], with correlation with downregulation of the UPF1 protein, we cannot exclude the possibility that in this case the treatment was insufficient.

Despite the fact that we did not successfully detect genes carrying a PTC using the GINI technology, we cannot rule out that the gene *INHBB *did not harbour a PTC since we were unable to sequence the full length of the gene. However, all other candidate genes were successfully sequenced without detecting a PTC. Therefore, we still believe that the siRNA mediated inhibition of the NMD machinery yields many false positive candidate genes.

In summary, we aimed to find candidate genes inactivated by nonsense mutation and deletion in the gastric cancer cell lines to further validate our results of DNA copy number profiling and expression analysis in primary gastric cancers. Although the GINI technology theoretically is a powerful method for identifying candidate tumour suppressor genes inactivated by nonsense mutations, siRNA mediated inhibition of the NMD machinery yielded false positive results in our hands. The GINI technique might be optimized by using a vector containing multiple small hairpin RNAs (shRNAs) that can silence multiple target sites simultaneously, to more effectively knockdown the NMD system[36]. Moreover, applying this strategy on multiple cell lines might be a more conservative approach applicable for selecting candidate genes, thereby contributing to less false positive test results. On the other hand, the discovery of new tumour suppressor genes inactivated by nonsense mutation by means of GINI may be redundant in the near future due to the emerging of the next-generation technologies in which the complete genome can be analyzed by massive parallel sequencing[37].

## Conclusion

The GINI technology by means of siRNA mediated inhibition of the NMD machinery in gastric cancer cell lines yielded false positive results in our hands.

## Abbreviations

NMD: nonsense-mediated decay; GINI: gene identification by nonsense mediated decay inhibition; CGH: comparative genomic hybridization; siRNA: small interfering RNA; shRNA: small hairpin RNA; PTC: premature termination codon; FDR: false discovery rate.

## Competing interests

The authors declare that they have no competing interests.

## Authors' contributions

TB performed the cell culture, siRNA transfections, RNA isolations, RT-PCR experiments and wrote the manuscript, MT performed the microarray experiments and sequence analysis, JE-B optimized all conditions for the cell culture, siRNA transfections and RT-PCR experiments, as well as guidance of the transfection procedure, AD provided the facilities to do the siRNA transfections and helped in coordinating the work, MvdW performed the microarray expression data analysis, BY provided the facilities for the microarrays experiments, GM and BC conceived and coordinated the study and helped in critically reviewing the manuscript. All authors read and approved the final manuscript.

## Pre-publication history

The pre-publication history for this paper can be accessed here:



## Supplementary Material

Additional file 1 **Primer sequences and conditions of the candidate genes**. This table shows the details of the primer sequences, annealing temperatures and extra PCR conditions of the candidate genes, inactivated by nonsense mutation and deletion, selected for mutation analysis. Genes sequenced for mutation analysis are shown in bold. Temp – annealing temperature. Click here for file
