# Supplementary material for: NMD inhibition fails to identify tumour suppressor genes in microsatellite stable gastric cancer cell lines
Source: BMC Med Genomics. 2009 Jun 29;2:39. doi: 10.1186/1755-8794-2-39 (PMC2709900; doi:10.1186/1755-8794-2-39)
Supplement: Additional file 1 — Primer sequences and conditions of the candidate genes. This table shows the details of the primer sequences, annealing temperatures and extra PCR conditions of the candidate genes, inactivated by nonsense mutation and deletion, selected for mutation analysis. Genes sequenced for mutation analysis are shown in bold. Temp – annealing temperature. [file 1755-8794-2-39-S1.doc]

Additional file 1

File format: DOC

Title: Primer sequences and conditions of the candidate genes.

Description: This table shows the details of the primer sequences, annealing temperatures and extra PCR conditions of the candidate genes, inactivated by nonsense mutation and deletion, selected for mutation analysis. Genes sequenced for mutation analysis are shown in bold. Temp - annealing temperature.

| **Gene** |  | **Sequence** | **Size** | **Temp** | **Remark** |
| --- | --- | --- | --- | --- | --- |
| **BMP5** | -96F | GCTACTGGGAAACTGTACCTC | 422bp | 55°C | 5% DMSO |
|  | +326R | GTCTCTTCTGCCAAGGATGC |  |  |  |
|  | +220F | CGTCCTCTGCACCTCTCTTT | 398bp | 55°C | 5% DMSO |
|  | +618R | TGTTGCTCCGGTCCTTGTAT |  |  |  |
|  | +513F | CACCAGCGAAGGCATTACAA | 416bp | 55°C | 5% DMSO |
|  | +929R | ACCTCACTCGCCTTGAAGAA |  |  |  |
|  | +807F | CAGCTCTGTGCAGAAACAGG | 433bp | 55°C | 5% DMSO |
|  | +1240R | CGTGGTCAGGAAACATCAGA |  |  |  |
|  | +1119F | CCAGAAGGATACGCTGCATT | 338bp | 55°C | 5% DMSO |
|  | +1455R | GGAAATTCCCCGTTTGTCTG |  |  |  |
| **DYM** | -142F | GACAGCGACTTCTCCTGACC | 434bp | 57°C | 5% DMSO |
|  | +292R | TGTGGTTCTGACATTCTGCTG |  |  |  |
|  | +204F | CCTCGAACAGGAAATCTTGG | 419bp | 55°C | 5% DMSO |
|  | +623R | AGACATGGACCTCGCATCAA |  |  |  |
|  | +546F | CTTTCCTGCCAACTCTTCCA | 421bp | 55°C | 5% DMSO |
|  | +967R | ATGAGGGGAAAGGACTGCTA |  |  |  |
|  | +844F | ACCAGAGTCTCCTGCTTCTG | 468bp | 57°C | 5% DMSO |
|  | +1312R | GACTCCCCAAGGAGATTTCA |  |  |  |
|  | +1212F | ACGGAAGATGATGGCTTCAAC | 433bp | 57°C | 5% DMSO |
|  | +1645R | GGTGAAGGGAATTTGTCAGG |  |  |  |
|  | +1528F | CGCTGAGTTCTAATGATGTTCC | 536bp | 57°C | 5% DMSO |
|  | +2064R | AGAACTTGAAGGGCTGCTTG |  |  |  |
| **MPP6** | -117F | TGGAGAAGAAAGGGATTTGG | 412bp | 55°C |  |
|  | +295R | CAATATCATGGGCCTCCAAC |  |  |  |
|  | +227F | TGTGGCAGAATTGGTTGGTA | 312bp | 55°C |  |
|  | +548R | ACCTCATGGCCATTGACTTC |  |  |  |
|  | +413F | GGAACCACTGGGTGTGACAT | 430bp | 55°C |  |
|  | +843R | CTCTTCCAGGAACTGGCTTG |  |  |  |
|  | +702 | CCTTGCAAAGAAGCAGGATT | 433bp | 55°C |  |
|  | +1135 | CCCTTGGTTTCCGTGAAGTA |  |  |  |
|  | +1045 | TAGGCCGAAGAAGCTTGAAA | 439bp | 55°C |  |
|  | +1484 | CGTGCACTTTCATCCACTGT |  |  |  |
|  | +1338 | TCAGAGTTTATGCCCTATGTGG | 409bp | 55°C |  |
|  | +1747 | CACCATAAAAATGCCTGGAC |  |  |  |
| **PLA2G4A** | -133F | CTCCGGAGCTGAAAAAGGAT | 303bp | 55°C |  |
|  | +170R | CTGCTGTCAGGGGTTGTAGA |  |  |  |
|  | +71F | TTACGTGCCACCAAAGTGAC | 480bp | 55°C |  |
|  | +551R | TGCAGAATGCAATCCTTCAC |  |  |  |
|  | +441F | TGGCTCTGTGTGATCAGGAG | 428bp | 55°C |  |
|  | +870R | GGTGACAGGTTGTCCAGAGC |  |  |  |
|  | +773F | TGTTAGCCACAATCCCCTTT | 490bp | 55°C |  |
|  | +1244R | GAGCCTCTGCTTTGTGAACC |  |  |  |
|  | +1121F | GGGAACAGTCGTTAAGAAGTA | 421bp | 55°C |  |
|  | +1542R | GTCCTGTGTGGCAAAGTCAC |  |  |  |
|  | +1407F | ATGGCCTTGGTGAGTGATTC | 455bp | 55°C |  |
|  | +1862R | TAGCACTCCTTCAGCCCTTC |  |  |  |
|  | +1752F | CCTCCGTTCAAGGAACTTCT | 555bp | 55°C |  |
|  | +2307R | TCATGGGATTGCAAACTGCC |  |  |  |
| **SLITRK6** | -134F | GTACGCAGTGGTTGGTGTTT | 403bp | 55°C |  |
|  | +269R | GCATTGGTAAGCCCAGAAAA |  |  |  |
|  | +186F | CCATCACGACCTTTCCAACT | 475bp | 55°C |  |
|  | +661R | CCCATTTGTTGTCCTCCAAC |  |  |  |
|  | +525F | CCTCCAAACATCTTCCGATT | 408bp | 55°C |  |
|  | +934R | TGGTGCTTTGGTGGGTAGTT |  |  |  |
|  | +849F | CATCTGGCAGCAACATCTTC | 425bp | 55°C |  |
|  | +1274R | TTGGTCAGGTGGTTACCATTT |  |  |  |
|  | +1098F | AAGCTCATTCTAGCGGGAAA | 500bp | 55°C |  |
|  | +1598R | TGTATCCATTGCTGCAGTCC |  |  |  |
|  | +1528F | CCCAGATTGACCTTGAGGAT | 436bp | 55°C |  |
|  | +1964R | TGCACAGGACTGTTGTCTCT |  |  |  |
|  | +1866F | TTCTGTGCTGCAGGGATAGT | 451bp | 55°C |  |
|  | +2317R | CTGTGATTCCCAGTTGCTGA |  |  |  |
|  | +2243F | CTTCCAAGATGCCAGCTCAT | 447bp | 55°C |  |
|  | +2690R | CCCAGTGATCCCTGAGTTTC |  |  |  |
| **CSTA** | -95F | GAGCTAGTGAACGCCTCTTT | 453bp | 55°C |  |
|  | +258R | TCAGCAAGGATCATGACTCAG |  |  |  |
| **KCNMB4** | -379F | ACAGAGAGACACCCGACGAG | 491bp | 57°C |  |
|  | +112R | GCAGAAGCCGAAGATGAAGA |  |  |  |
|  | +34F | CTTCATCTTCGGCTTCTGCT | 410bp | 57°C |  |
|  | +444R | GCAAGTAAATGGCTGGGAAC |  |  |  |
|  | +336F | TGCTCCTATATCCCTCCCTGT | 474bp | 57°C |  |
|  | +792R | TCCAGTTGTGCCTGTTTCTG |  |  |  |
| **PTPRJ** | -129F | AGGAGGAGGCGAAGGAGA | 442bp | 55°C | 5% DMSO |
|  | +334R | TTTGAGATGCCCCATCAGTC |  |  |  |
|  | +259F | GTGAAAGCTCTGGAGCCAAC | 418bp | 55°C |  |
|  | +676R | AGAGAGCAGCCTTCCTCACA |  |  |  |
|  | +553F | CCATCACTCCAGGAATAGGC | 452bp | 55°C |  |
|  | +1007R | TCTAACCCGACAAGCAGGAC |  |  |  |
|  | +917F | CCCTGTGCATGATGAGTCC | 476bp | 55°C |  |
|  | +1393R | CCACTGTCACTCGGAAGTCA |  |  |  |
|  | +1273F | GCTCCAGCACCTTCTACAACA | 412bp | 55°C |  |
|  | +1685R | CTCTTCCAGTCCAGCCACAT |  |  |  |
|  | +1586F | GGCATCTCGGACAGTTTGCAA | 436bp | 55°C | 5% DMSO |
|  | +2022R | GACTACTTGTGTTGCGTTGC |  |  |  |
|  | +1987F | CAACACCACAGCAGCAACTT | 417bp | 55°C |  |
|  | +2324R | TCAGTGCCATTCTCAGAGGA |  |  |  |
|  | +2237F | CAATGCAGGCTTTGAGCTG | 455bp | 55°C |  |
|  | +2691R | GCTCTGAGAACGTCCCTTTT |  |  |  |
|  | +2587F | CTGCAGATGTCCTGAAATACA | 399bp | 55°C |  |
|  | +2987R | AGAAGATGAAGCCTCCCACA |  |  |  |
|  | +2874F | CGCTACTCAGATGCTGTTTC | 477bp | 55°C |  |
|  | +3351R | CGGTAAAGGTCCTTGTGTGG |  |  |  |
|  | +3259F | AGACCCATTCAACGGATGAC | 407bp | 55°C |  |
|  | +3666R | AACGAGGTACCGGAAGTTGA |  |  |  |
|  | +3576F | CACCCTCTGAGACAGTTCCA | 476bp | 55°C |  |
|  | +4052R | ACGGTCTGGTTCACTCCAGA |  |  |  |
| **SNN** | -78F | GTGTCCAGCCTGAGTTCCAG | 448bp | 57°C | 5% DMSO |
|  | +370R | AGTGTCAGCCCTTCCTCTCA |  |  |  |
| **INHBB** | -327F | TGGGGAGAAGGCTGCAGAT | 500bp | 55°C, 57°C | no fragment |
|  | +173R | CACGACGTACAGGTGTCCT |  |  |  |
|  | +36F | TGCCTTCTGCTGCTGGCG | 465bp | 55°C, 57°C | no fragment |
|  | +501R | GCCTTCGTTGGAGATGAAGA |  |  |  |
|  | +415F | GCGTTTCCGAAATCATCAGC | 425bp | 57°C | 7.5% DMSO |
|  | +840R | AGCCTGCACCACCACAAAG |  |  |  |
|  | +732F | CGACTCAACCTAGACGTGCA | 413bp | 55°C |  |
|  | +1145R | ATGGACATGGTGCTCAGCTT |  |  |  |
|  | +963F | ATAGCACCCACCGGCTACTA | 465bp | 57°C | 5% DMSO |
|  | +1428R | CAGTTGAGGGTTCTCGCTCT |  |  |  |
| **TXNL4B** | -132F | GCTGATGAAAGGAGCTGTCC | 447bp | 57 °C |  |
|  | +315R | GCTTCCCACAAACTTAGTGTG |  |  |  |
|  | +178F | TGGTGGATGTGGACCAAACT | 437bp | 55°C |  |
|  | +615R | AACCAGTGGGGTCTTTTCCT |  |  |  |
| FOXK1 | -5F | CGAACATGGCCGAAGTCGG | 440bp |  |  |
|  | +435R | CGAGATGAAGCTGGACAGG |  |  |  |
|  | +336F | GAGTTCGAGTTCCTCATGCG | 475bp |  |  |
|  | +811R | TCTGCACAAAGCGGTAACTG |  |  |  |
|  | +740F | CATCAGTGTCCCCAACTCCT | 417bp |  |  |
|  | +1157R | TCAGAGGCAGGGTCTATTCG |  |  |  |
|  | +1055F | TATCCGGCACAACCTCTCTT | 468bp |  |  |
|  | +1523R | GGCTGCTGTGAGGTTACGAT |  |  |  |
|  | +1339F | TTCCACACGACCCTGAGTTT | 424bp |  |  |
|  | +1763R | CACCGTCTGGATGACTCCAC |  |  |  |
|  | +1699F | TGGAGGAGAAACCCACCAT | 450bp |  |  |
|  | +2149R | GTGTGGTTACAGCCCCACT |  |  |  |
|  | +1888F | TTCCCACGAACAGTTTAGCC | 497bp |  |  |
|  | +2385R | TGTCTTAAACCACGGGAAGG |  |  |  |
